# Assessing Postzygotic Isolation Using Zygotic Disequilibria in Natural Hybrid Zones

**DOI:** 10.1371/journal.pone.0100568

**Published:** 2014-06-20

**Authors:** Xin-Sheng Hu, Francis C. Yeh

**Affiliations:** 1 Department of Plant Sciences, University of Oxford, Oxford, United Kingdom; 2 Department of Renewable Resources, University of Alberta, Edmonton, Canada; Macquarie University, Australia

## Abstract

Hybrid zones as windows on evolutionary processes provide a natural laboratory for studying the genetic basis and mechanisms of postzygotic isolation. One resultant pattern in hybrid zones is the Hardy-Weinberg disequilibrium (HWD) for a single locus or the linkage disequilibrium (LD) for multiple loci produced by natural selection against hybrids. However, HWD and the commonly used low-order gametic or composite digenic LD cannot fully reflect the pattern of the high-order genotypic interactions. Here we propose the use of zygotic LD to elucidate the selection mechanisms of postzygotic isolation, and its calculation is based on genotypic frequencies only, irrespective of the type of mating system. We numerically and analytically show that the maximum composite digenic LD is always greater than the maximum absolute zygotic LD under the linear-additive selection, but is comparable to or smaller than the maximum absolute zygotic LD under the strong epistatic selection. Selection mechanisms can be inferred by testing such differences. We analyze a previously reported mouse hybrid zone assayed with genome-wide SNPs, and confirm that the composite digenic LD cannot appropriately indicate all possible significant genotypic interactions for a given SNP pair. A large proportion of significant zygotic LDs, ∼75% in general in the mouse hybrid zone, cannot be revealed from the composite digenic LD analysis. Statistical tests indicate that epistatic selection occurred among multiple loci in the mouse hybrid zone. The results highlight that the joint patterns of the composite digenic and zygotic LDs can help to elucidate the selection mechanism that is potentially involved in postzygotic isolation.

## Introduction

Postzygotic isolation occurs when the hybrids produced by two genetically diverging species in sympatry are successfully formed but eventually turn out to be inviability or sterility [1]. In flowering plants, this takes place in the sporophyte stage where ovules of one species and pollen from the other species are fused to produce zygotes, irrespective of the presence or absence of prezygotic isolation. The genetic mechanism for postzygotic isolation may come from the antagonistic effects either within loci (between different alleles of a locus; e.g., low heterozygote fitness) or among loci (e.g., the asymmetric genic incompatibility among loci; [1], [2], [3], [4]), or from both. Alternatively, the genetic mechanism may come from the effects of ecological factors that induce antagonistic interaction within or between loci, resulting in hybrid inviability or sterility [1]. As a consequence, an observable pattern for a single locus is the likely significant Hardy-Weinberg disequilibrium (HWD) due to heterozygous deficiency in the hybridizing populations. An observable pattern for multiple loci is the likely significant low- and/or high-order linkage disequilibrium (LD) among linked or unlinked loci [5]. HWD measures the variation at individual single loci while LD measures the association between loci. Use of LD to characterize and reveal reproductive isolation in natural hybrids is an important perspective for insights into the mechanism of postzygotic isolation [6], [7], [8]. The current availability of genome-wide single nucleotide polymorphisms (SNPs) provides us with an opportunity to use genome-wide pattern of LD to study the genetic basis and mechanisms of speciation [9], [10], [11], [12], [13].

Previous relevant theory mainly emphasizes the use of low-order gametic LD to characterize the genetic mechanisms of maintaining natural hybrid zones [14], [15]. Its analysis relies on the assumption of Hardy-Weinberg equilibrium (HWE) or random mating in the naturally hybridizing populations, which otherwise cannot yield the estimate of gametic LD from diploid genotyping data [16]. This assumption could be violated in the hybridizing populations since inbreeding and other processes (e.g., migration and selection) can cause HWD during the process of gene introgression. Recently, Teeter et al. [9] used a composite digenic LD to detect Dobzhansky-Muller incompatibility model for reproduction isolation in a house mouse hybrid zone (*Mus musculus* ×*M. domesticus*). This measure removes the assumption of HWE or random mating, and hence effectively removes the errors of estimating gametic LD from diploid genotyping data. However, one crucial issue of this analysis is that the composite digenic LD (low-order) confounds the information of multiple genotypic interactions [16] and cannot explicitly specify the genotypic interactions that are potentially associated with reproductive isolation. This is the same case for the use of gametic LD or the use of HWD in a single locus. Furthermore, the composite digenic LD recovers the gametic LD under HWE [16], [17].

Here, we propose the use of zygotic LD, genetically related to but conceptually different from the composite digenic or gametic LD, to characterize the genetic mechanism of postzygotic isolation. Zygotic LD is termed as the difference between the joint genotypic frequency at two loci and the product of genotypic frequencies at each locus [18], [19], [20], [21], [22], [23], or as the covariance of genotypic frequencies at two loci. Like the composite digenic LD, this measure removes the assumption of HWE or random mating. Previous studies of zygotic LD lie in the aspects of the effects of partial inbreeding [24] or a mixed mating system [25], the proposition of testing selection in an artificial population [18], [19], the relevant conceptual insights [26], [27], the statistical issue on estimating zygotic LD [22], [23], [28], the potential application of zygotic LD to mapping quantitative trait loci (QTL; [29]), and the discussions of zygotic LDs for elucidating evolutionary processes [30], [31]. Hu [32] further shows that zygotic LD is more informative than gametic LD in detecting natural population history in a continent-island model. So far, this measure has not been applied to detecting the genetic mechanisms of reproductive isolation in hybrid zones except the use of cytonuclear genotypic LD, a conceptual analogy to zygotic LD [33], [34]. Thus, it is of interest to associate zygotic LD with natural selection in hybrid zones.

In this study, we consider a dispersal-dependent hybrid zone where gene flow is involved in producing the spatial pattern of zygotic LD. In flowering plants, this can be mediated by seed flow that directly generates zygotic LD, or pollen flow that directly generates the composite digenic or gametic LD but indirectly affects zygotic LD. Under this background, we concentrate on how different models of natural selection against heterozygotes change the pattern of zygotic LD, and compare the similarity and difference between the composite digenic and zygotic LDs. Two models of selection are examined: a linear-additive viability model and epistatic selection. In the linear-additive viability model, cumulative selection from multiple loci could further lower hybrid fitness, reinforcing reproductive isolation. In the epistatic selection model (e.g., Dobzhansky-Muller incompatibility model; [2], [3]), genes from distinct parents antagonistically interact to lower hybrid fitness, which also reinforces reproductive isolation. Thus, it is important to elucidate these two distinct selection mechanisms in maintaining natural reproductive isolation, analogous to the significance of elucidating intrinsic and extrinsic selection against hybrids [35], [36]. Both selection processes can change zygotic LD [32]. Here, we further show that the joint spatial patterns of the composite digenic and zygotic LDs can aid in inferring distinct selection processes.

In the following sections, we begin by using both simulation and analytical approaches to compare the patterns of the composite digenic versus zygotic LDs under the linear-additive or the epistatic selection in a hybrid zone. This provides a theoretical basis for inferring selection mechanisms in a hybrid zone. We then provide a statistical method to test the difference between composite digenic and zygotic LDs. We finally analyze a natural mouse hybrid zone examined by Teeter et al. [9]. This hybrid zone was generated after *M. domesticus* moved into Western Europe in the last 3000 yr [37]. The mouse hybrid zone has been studied in six transects (for details, see reviews by Teeter et al. [9]), and is a tension zone (intrinsic selection against hybrids; [38]). Both autosomes and sex chromosomes are involved in postzygotic isolation. Teeter et al. [9] investigated the genome-wide gene flow across the hybrid zone in Bavaria, Germany, and interpreted that the Dobzhansky-Muller's incompatibility model was responsible for reproductive isolation. Here, we demonstrate that the joint patterns of low- and high-order LDs can elucidate the potential selection mechanisms of speciation mediated in the form of a hybrid zone.

## Results

### Simulation Comparison

#### Methodology

Simulation is based on one dimensional stepping-stone model by assuming the same effective population size (*N_e_*) for each population of a hermaphrodite plant species [15], [35], [39]. Consider two diallelic loci, with alleles *A* and *a* at locus A and *B* and *b* at locus B; and the recombination rate between them is *r*. Initially, all populations at the left side (*x*<0) of a midpoint (*x* = 0) are fixed by *AABB*, while all populations at the right side (*x*>0) are fixed by *aabb*. These distinct gene pools meet through pollen and seed dispersal and produce a hybrid zone. A constant proportion of pollen grains, *m_P_*/2, and seeds, *m_S_*/2, are exchanged between two adjacent neighbors for each population. Each population follows the same life cycle: pollen and ovules generation, pollen flow, random mating and seed generation, seed flow, natural selection in the sporophyte stage, and genetic drift. Mutation effects and selection in the gametophyte stage are excluded.

In an ecological hybrid zone (extrinsic postzygotic isolation), homozygote *AABB* is more favorable than heterozygotes at the left side (*x*<0) while *aabb* is more favorable at the other side (*x*>0), i.e. the simplest model initially addressed by Haldane [40] in a cline theory. Two selection regimes are considered. The first selection regime is a linear-additive viability model. Let Wrightian fitnesses 1+*s*_1_ *g*_1_, 1+*h*_1_ *s*_1_ *g*_1_, and 1-*s*_1_ *g*_1_ for genotypes *AA*, *Aa*, and *aa*, and 1+s_2_g_1_, 1+*h*_2_ *s*_2_ *g*_2_, and 1-*s*_2_ *g*_2_ for *BB*, *Bb*, and *bb* in a population at position *x* (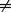
0), respectively. *h*_1_ and *h*_2_ are the degrees of dominance at loci A and B, respectively; and *g*_1_ and *g*_2_ are the function indicating the pattern of environment-dependent selection at loci A and B, respectively. Let *g*_1_or *g*_2_ = 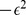 when *x*>0, and 1 when *x*<0 [40], where 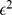 reflects the relative selection intensity between two sides.

The second selection regime in the ecological zone includes epistatic selection. Let 1+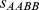 be the fitness of genotype *AABB,* and 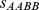 is decomposed as 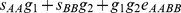 where 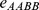 is the epistatic selection part. Fitness for other two-locus genotypes can be set in the way similar to setting the fitness for *AABB*. Here, we assume that 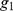
and 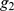 are concordant in sign and that the epistatic selection induced by environmental factors has negative effects on hybrid fitness.

In a tension zone (intrinsic postzygotic isolation), two selection regimes are considered as well. The first selection regime is the linear-additive viability model. Let 1, 1-*s*_1_, and 1 be the fitness of genotypes *AA*, *Aa*, and *aa*, and 1, 1-*s*_2_, and 1 be the fitness of *BB*, *Bb*, and *bb* in a population at position *x*, respectively [41]. The fitness for any two-locus genotype can be calculated by multiplying the fitness of individual genotypes at each locus.

The second selection regime includes epistatic selection. Let 1+*s_AaBB_* be the fitness of genotype *AaBB*. The selection coefficient *s_AaBB_* is decomposed as 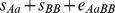 where *e_AaBB_* (≠0) is the epistatic component, *s_Aa_* (≠0) is the additive part of *s_AaBB_* for genotype *Aa*, and *s_BB_* ( = 0) is the additive part of *s_AaBB_* for genotype *BB*. Fitness for other two-locus genotypes can be set in a similar way. The epistatic selection arises from the interaction between distinct genetic backgrounds.

Let *D_AABB_* be the zygotic LD between *AA* at locus A and *BB* at locus B, which is calculated by
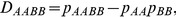
(1)where *p_AABB_*, *p_AA_*, and *p_BB_* are the frequencies of genotypes *AABB*, *AA*, and *BB*, respectively [22], [23]. Note that zygotic LD in Eq. (1) is conceptually and quantitatively different from the quadrigenic LD whose calculation relies on gametic LD estimated from diploid genotyping data under the assumption of HWE or random mating [16], [42]. Zygotic LD for any other genotypes can be defined in the same way as Eq. (1). For a pair of diallelic loci, there are eight zygotic LDs, but only four of them (*D_AABB_*, *D_AABb_*, *D_AaBB_*, and *D_AaBb_*) are independent [32]. Thus, we mainly concentrate on these four independent zygotic LDs.

Let *Δ_AB_* be the composite digenic LD. From Weir ([16], p.126), the composite digenic LD can be expressed in terms of zygotic LD:
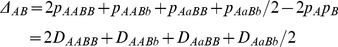
(2)


The composite digenic LD is the sum of the four zygotic LDs with unequal coefficients. The difference of *Δ_AB_* from gametic LD, *D_AB_*, lies in that *Δ_AB_* includes the additional associations of alleles sampled between individuals [16].

Simulations are conducted in the following steps. Given a set of parameters (*N_e_*, *m_S_*, *m_P_*, and *s*'s), simulation starts from adult populations and proceeds according to the life cycle. Assume that gametic frequencies in migrating pollen and genotypic frequencies in migrating seeds are the same as those in the source populations. After natural selection in the sporophyte stage, the numbers of two-locus genotypes are randomly sampled according to the multinomial distribution of genotypic frequencies in each population of the effective size *N_e_*. Random numbers with uniform distribution within (0, 1) for the sampling purpose are generated using the routine of Press et al. ([43], pp. 210–211). Five thousand independent simulation runs are conducted in each case, and replicates are used to calculate means and standard deviations of zygotic and composite digenic LDs. Parameter settings are arbitrary as long as they are biologically meaningful and a steady-state distribution can be eventually reached.

#### Ecological zone

**Figure 1:**
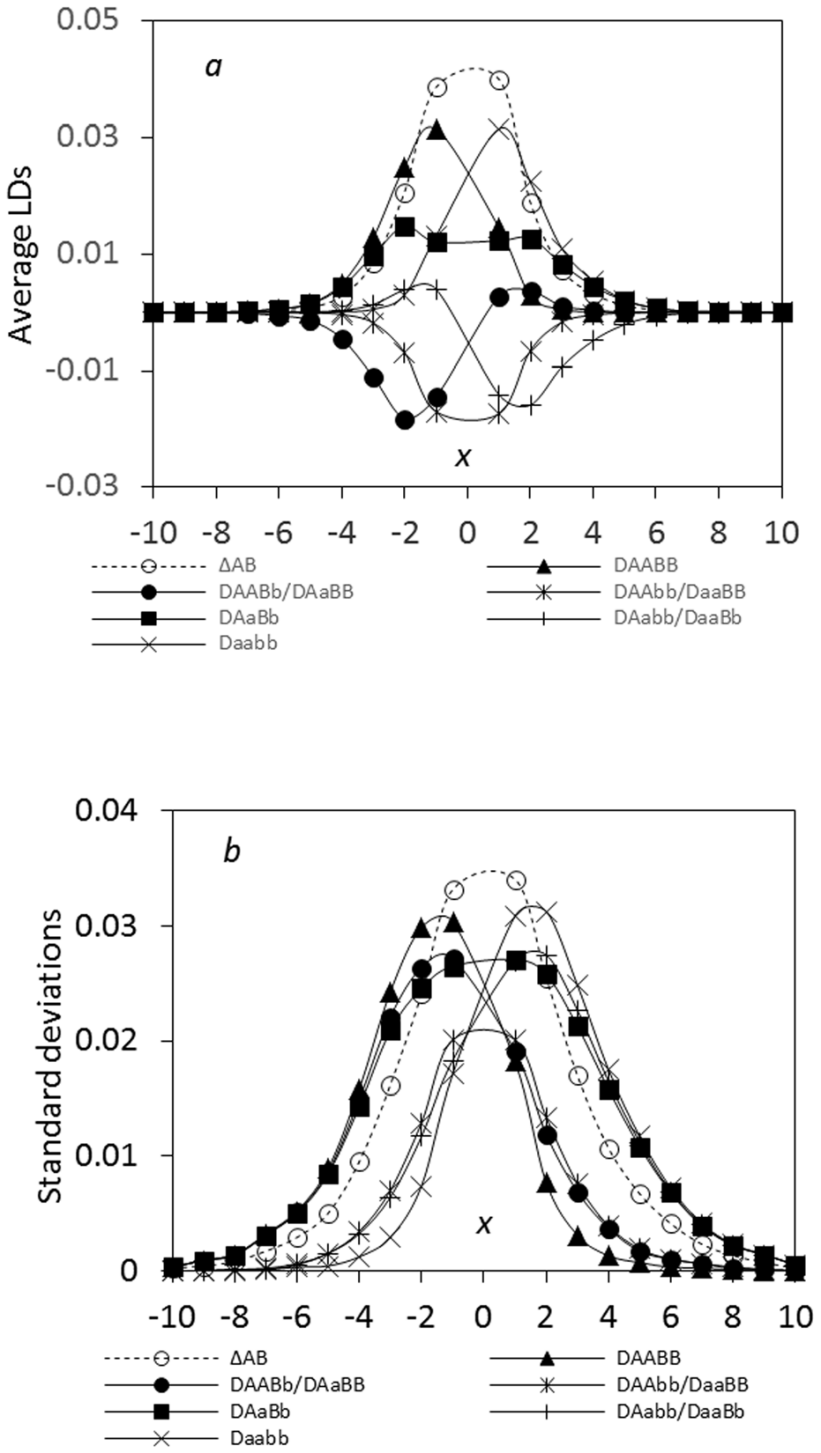
Comparison of the steady-state composite digenic and zygotic LDs in an ecological zone: a. the average LDs; b. the standard deviations. Results are obtained from 5000 independent simulation runs. Parameter settings are the migration rate of pollen *m_P_* = 0.08 and seeds *m_S_* = 0.04, the recombination rate *r*  = 0.1, the selection coefficient *s*_1_ = *s*_2_ = 0.02, the relative selection intensity 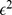 = 1.0, and the effective population size *N_e_* = 100.

Under the linear-additive selection, distinct spatial patterns exist among the composite digenic and zygotic LDs across a hybrid zone. First, the maximum composite digenic LD is always greater than the maximum absolute zygotic LD (Figure 1A and B), i.e. max. |*Δ_AB_*|> max. |
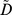
|, where 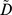
is one of the four zygotic LDs (*D_AABB_*, *D_AABb_*, *D_AaBB_*, *D_AaBb_*). Second, the composite digenic LD has the maximum in the vicinity of zone center. Zygotic LDs exhibit discordant patterns among different genotypes. The homozygote-homozygote genotypes (*AABB*, *aabb*, *AAbb*, and *aaBB*) have one maximum at the zone central region. The homozygote-heterozygote genotypes (*AABb*, *AaBB*, *Aabb*, and *aaBb*) have a minimum (negative) value at one side of the zone and a small peak (positive) at the other side. The heterozygote-heterozygote genotype (*AaBb*) may exhibit one peak at the center region for closely linked loci or two peaks for loosely linked loci. Third, the standard deviations of the composite digenic and zygotic LDs are generally consistent with the patterns of their absolute means, with larger standard deviations in the regions of maximum or minimum zygotic LDs (Figure 1B).

Extensive simulations indicate the above three features hold under different extents of seed and pollen flow or genetic drift. The difference is that large seed and pollen flow can expand both zygotic and composite digenic LDs in more populations away from the zone center. A large genetic drift effect (smaller population size) can increase variations (data not shown here).

**Figure 2:**
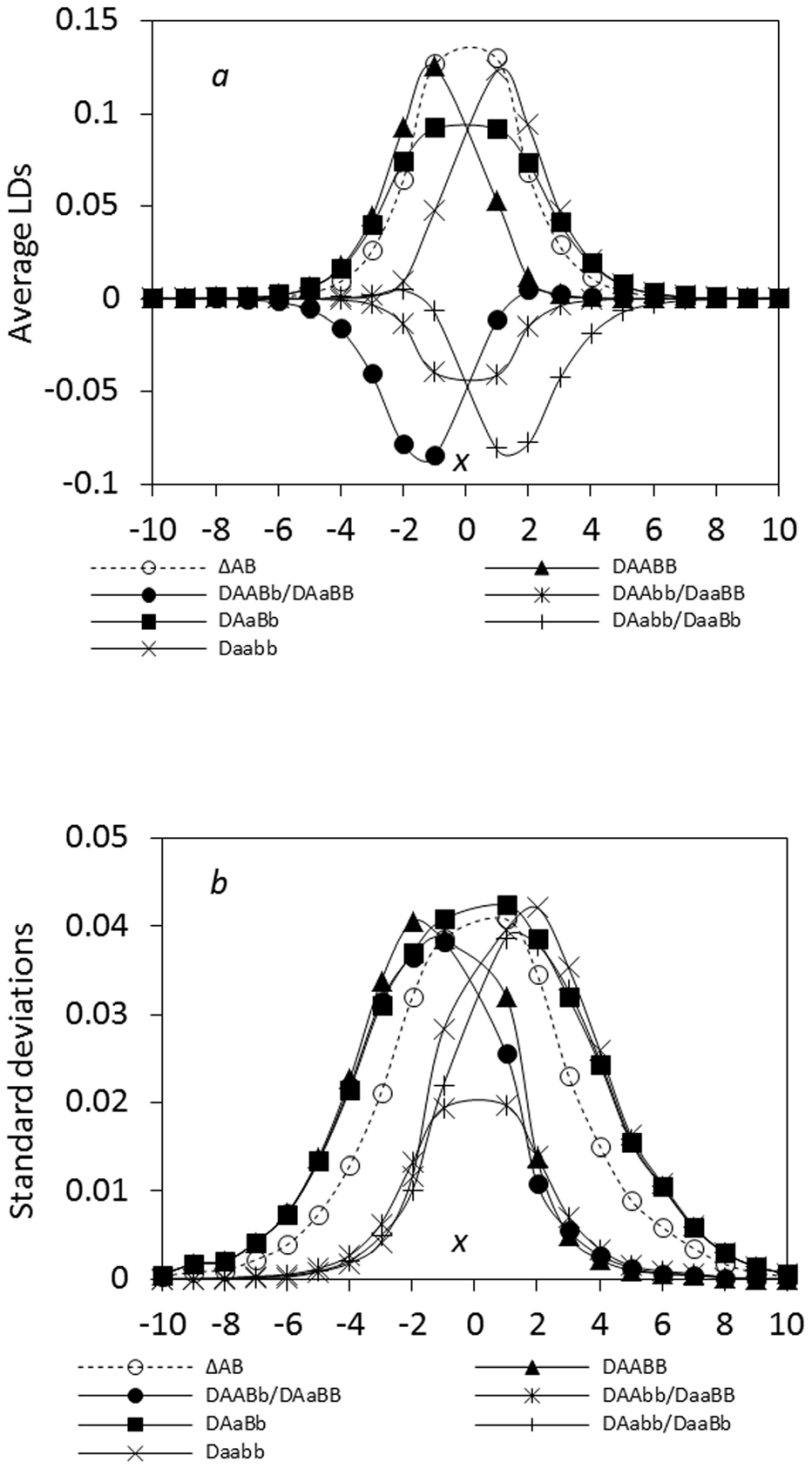
Epistatic effects on the steady-state composite digenic and zygotic LDs in an ecological zone: a. the average LDs; b. the standard deviations. Results are obtained from 5000 independent simulation runs. The additive selection parts are set as *s_AA_* = *s_BB_* = 0.02, *s_Aa_* = *s_Bb_* = 0, and *s_aa_* = *s_bb_* = −0.02. The epistatic parts are *e_AABB_* = 0, *e_AABb_* = −0.01, *e_AAbb_* = −0.02, *e_AaBB_* = −0.01, *e_AaBb_* = −0.02, *e_Aabb_* = −0.01, *e_aaBB_* = −0.02, *e_aaBb_* = −0.01, and *e_aabb_* = 0. Other parameters are the migration rate of pollen *m_P_* = 0.08 and seeds *m_S_* = 0.04, the relative selection intensity 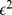 = 1.0, the recombination rate *r* = 0.02, and the effective population size *N_e_* = 100.

Under the epistatic selection, a crucial feature is that the maximum zygotic LD is comparable to or greater than the maximum composite digenic LD. Figure 2 shows the patterns under epistatic selection, with the same order of strengths as the additive selection in Figure 1. The maximum zygotic LDs for parental genotypes *AABB* ( = 0.1250) and *aabb* ( = 0.1227) are very close to the maximum composite digenic LD ( = 0.1262; Figure 2A). Their standard deviations are generally consistent with the patterns of their absolute means, with larger standard deviations in the regions of the maximum or minimum zygotic LDs (Figure 2B). Strong epistatic selection can further increase the maximum zygotic LDs (data not shown here). Effects of other driving forces (migration and genetic drift) do not alter this pattern.

#### Tension zone

**Figure 3:**
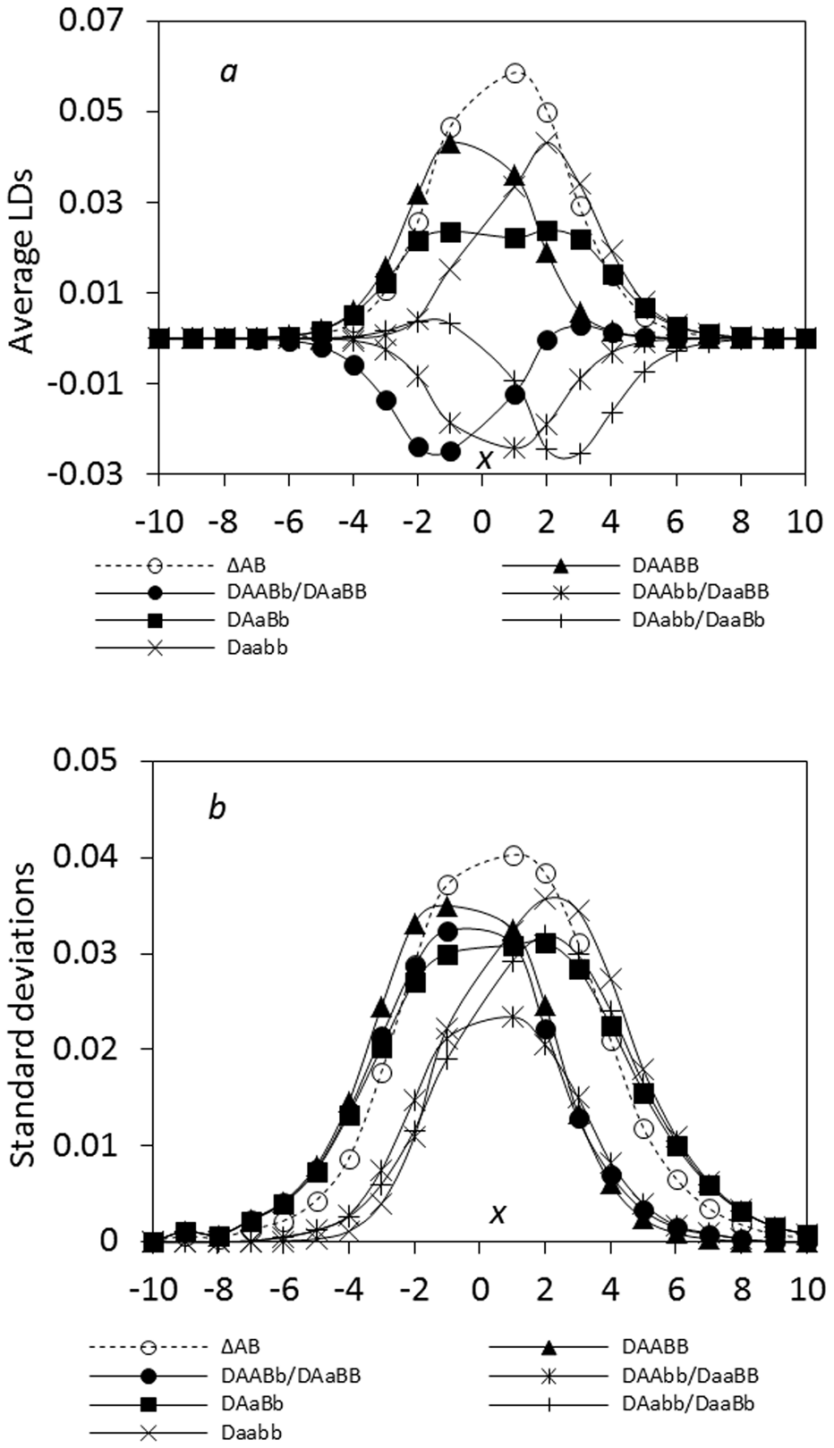
Comparison of the steady-state composite digenic and zygotic LDs in a tension zone: a. the average LDs; b. the standard deviations. Results are obtained from 5000 independent simulation runs. Parameter settings are the migration rate of pollen *m_P_* = 0.08 and seeds *m_S_* = 0.04, the recombination rate *r* = 0.05, the selection coefficient *s*_1_ = *s*_2_ = 0.02, and the effective population size *N_e_* = 100.

Under the linear additive selection, the maximum composite digenic LD is always located at the zone center (*p* = 1/2), different from the case in the ecological zone (max.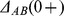 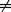
max.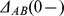; [36]). The three features observed in the ecological zone (Figure 1) remain present under the effects of seed and pollen flow, genetic drift, and recombination rate. The initial parental genotypes (*AABB* and *aabb*) have one peak near the zone center (Figure 3). The recombinant genotypes at one locus (*AABb*, *AaBB*, *Aabb*, and *aaBb*) have a minimum at one side of the zone but a small peak at the other side. The double heterozygote genotype (*AaBb*) can exhibit one peak under tight linkage or two symmetric peaks under loose linkage. The standard deviations are large at the regions of the maximum or minimum LDs (Figure 3B).

**Figure 4:**
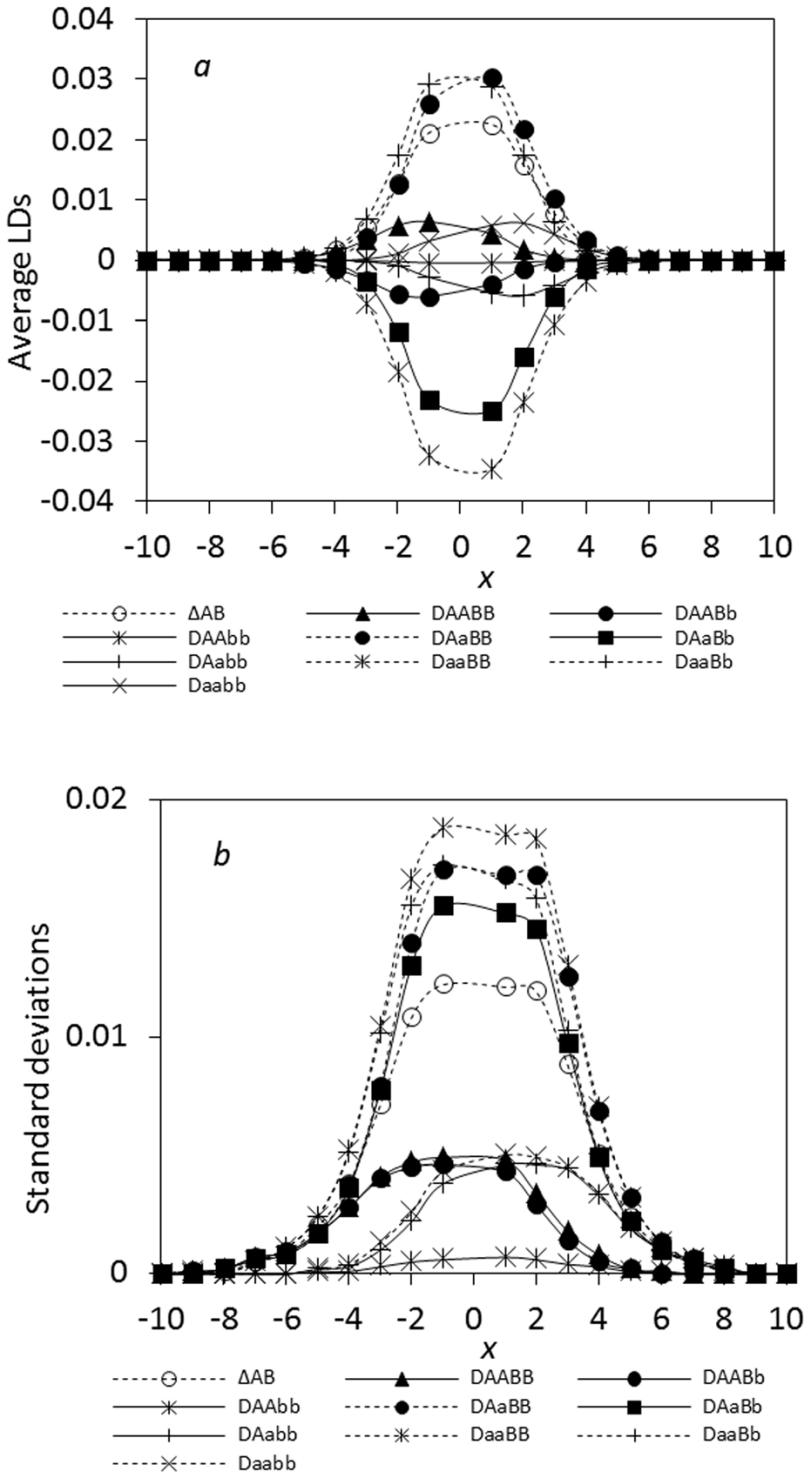
Epistatic effects on the steady-state composite digenic and zygotic LDs in a tension zone: a. the average LDs; b. the standard deviations. Results are obtained from 5000 independent simulation runs. The genotypic fitness is set as 1 for *AABB* and *aabb*, 0.99 for *AaBB* and *aaBb*, 0.5 for *AABb* and *Aabb*, 0.98 for *aaBB*, 0.5 for *AAbb*, and 0.5 for *AaBb*. Other parameters are the migration rate of pollen *m_P_* = 0.2 and seeds *m_S_* = 0.1, the recombination rate *r* = 2%, and the effective population size *N_e_* = 100.

Under the epistatic selection, we examine the antagonistic interactions between distinct genetic backgrounds. Similar to the results in the ecological zone, the maximum zygotic LD becomes comparable to the maximum composite digenic LD as the strength of epistatic selection increases (data not shown here). Under strong epistatic selection, the maximum zygotic LD can be greater than the maximum composite digenic LD. For instance, consider the same setting as matrix (14) of Gavrilets [4], i.e. the Dobzhansky-type epistatic selection [2]. Alleles *A* and *b* are assumed to have large negative interactions on fitness. The maximum average zygotic LD is |*D_aaBB_* | = 0.0346; while the maximum average composite digenic LD is *Δ_AB_* = 0.0226 (Figure 4A). All standard deviations of zygotic and composite digenic LDs are generally large (or small) in the regions of large (or small) LDs (Figure 4B).

The above simulations indicate that the maximum composite digenic LD is always greater in magnitude than the maximum zygotic LD (high-order) in a hybrid zone under the linear-additive selection. The maximum composite digenic LD is smaller in magnitude than or comparable to the maximum zygotic LD under epistatic selection.

### Analytical Comparison

Here we analytically compare zygotic and composite digenic LDs arising from the linear-additive selection. A diffusion process is used to approximate gene flow in natural hybrid zones [36]. *m_S_* and *m_P_* are identical to the dispersal variances of seeds, 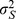, and pollen, 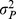, respectively [44]. Mutation and genetic drift effects are excluded. Weak selection is assumed so that the terms containing the second or higher orders of selection coefficients are negligible. Migration rate with the order similar to selection coefficients is considered so that a balance between selection and migration effects can occur for individual loci. According to the life cycle (without genetic drift), Hu [36] derives the recursion expressions for genotypic frequencies in both ecological and tension zones, which can be applied to calculating the composite digenic and zygotic LDs.

#### Ecological zone

Let 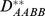 be the zygotic LD for genotype *AABB* after selection in the sporophyte stage, and 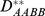 =  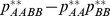 where 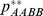, 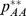 and 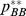 are the genotypic frequencies after selection for *AABB*, *AA*, and *BB*, respectively. For simplicity, use the notation of 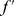
( = 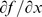) and
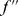
( = 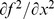) for a function *f*. From Hu [36], the recursion equation for 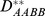 within a time interval, 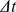, for the population at position *x* is derived as
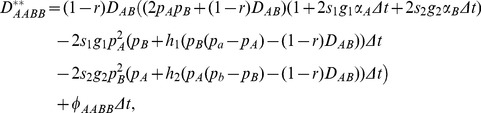
(3)where the change due to gene flow, 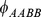, is
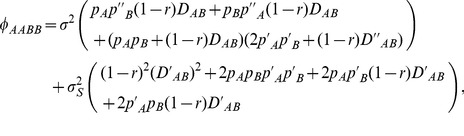
(4)in which 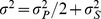, 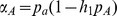, 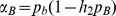, 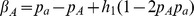, and 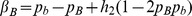. In Eq. (3), the factor (1-*r*) times 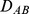 is because we start to set gametic LD in the preceding adults (one generation difference between adults and pollen/ovules; [36]).

The recursion equations for 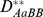, 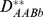, and 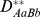 can be derived in a similar way, but are not detailed here.

Without loss of comparing zygotic and composite digenic LDs, we consider the coincident clines between two loci, which enhances reproductive isolation and genomic cohesion [35], [45]. Let *s*_1_ = *s*_2_ = *s*, *h_1_* = *h_2_* = *h*, *g*_1_ = *g*_2_ = *g*, *p_A_* = *p_B_* = *p*, and *q* = 1-*p* for a population at position *x*. According to Hu [36], 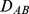, can be approximated by 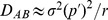, which gives good agreement with the true value in the case of 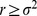 or even in the presence of epistasis [46]. Note that in the preceding approximation, the condition of 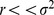 could cause 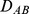 be greater than 1/4.

Under the coincidence of gene frequencies and *h* = 0 [36], we can obtain: 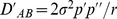, 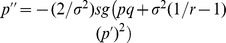, 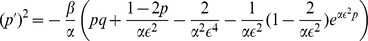, for *x*>0, and 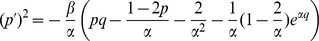, for *x*<0, where 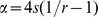 and 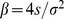. The boundary allele frequency at *x* = 0, denoted by *b_0_*, can be calculated from 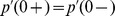 using an iterative approach [40], i.e.
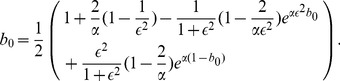
(5)


Under the coincidence of gene frequency clines, the steady-state zygotic LD in a population at position *x* is simplified as:
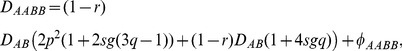
(6)where the migration component is
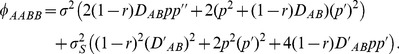
(7)


Both migration and selection can contribute to zygotic associations.

*D_AaBB_* equals *D_AABb_* due to the symmetry between the two loci under the coincident gene frequencies, but is unequal to *D_AABb_* under the non-coincidence of gene frequencies across a hybrid zone. The steady-state *D_AaBB_* is derived as
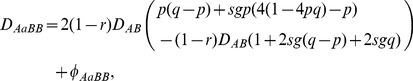
(8)where the migration component is
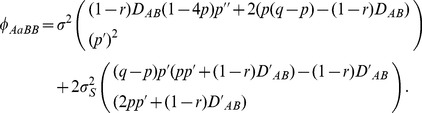
(9)


The steady-state zygotic LD for double-heterozygote genotype, *D_AaBb_*, is derived as
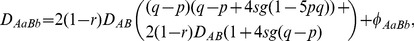
(10)where the migration component is 
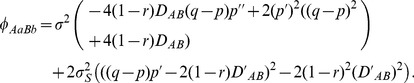
(11)


Under the case of coincident clines, the steady-state composite digenic LD is derived as
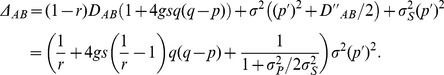
(12)



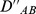
 in the above expression is omitted because it is of the order similar to the selection coefficient. Both selection and migration can contribute to an inequality between *Δ_AB_* and *D_AB_*. At the boundary point, *Δ_AB_* is spatially interrupted, i.e. 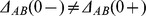 [36]. The maximum composite digenic LD can occur at the zone center if the boundary gene frequency *b_0_* equals 0.5, which otherwise may occur at either side of the zone center. This is because the boundary gene frequency *b_0_* can be altered by 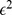, *r*, and *s*. Seed flow makes the composite digenic LD be greater than the gametic LD, while pollen flow makes them be close to each other.

**Figure 5:**
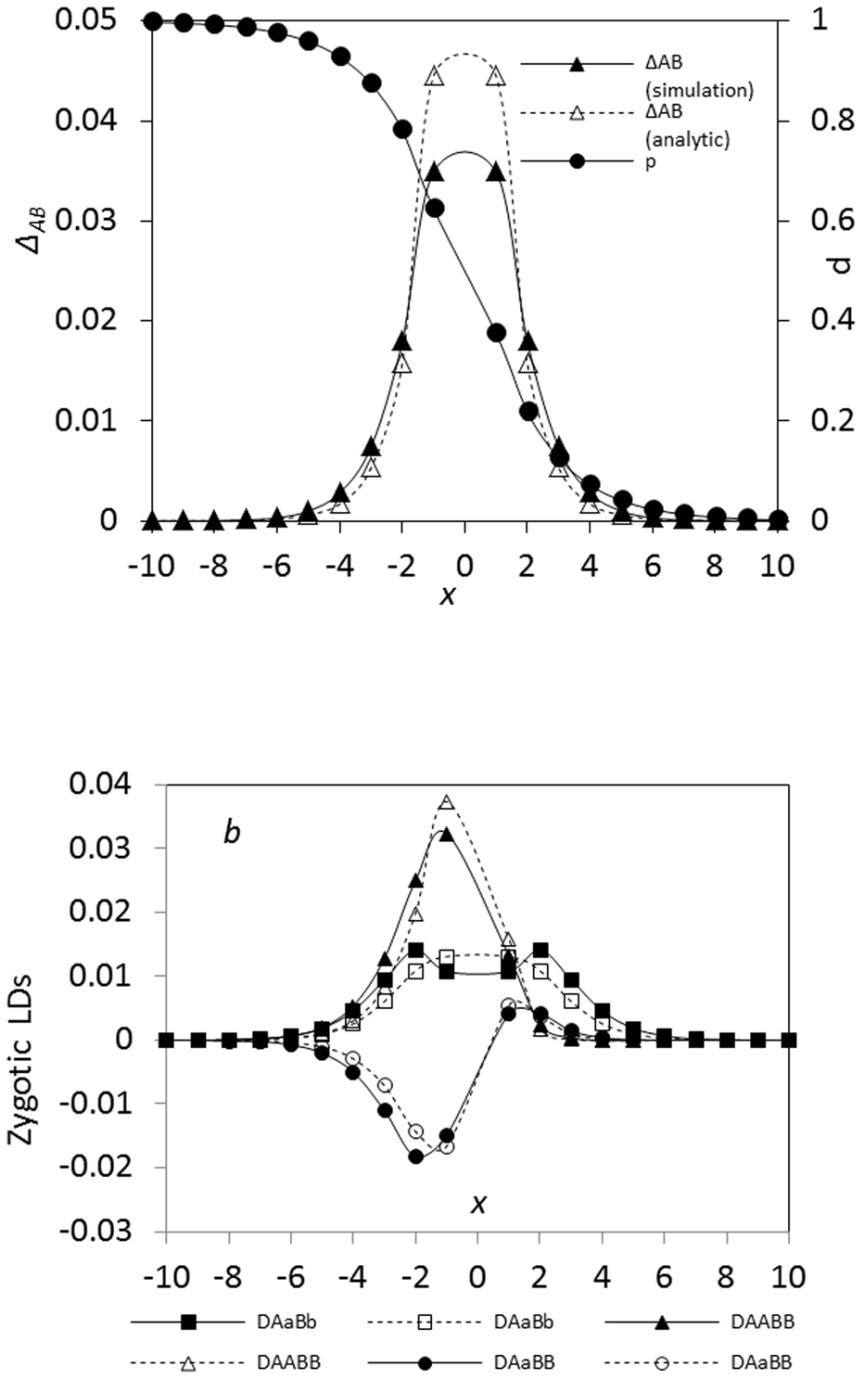
Comparison between the analytical model and simulation results in an ecological zone (no genetic drift effects). In (a) and (b), the dashed lines represent the results of the analytic model, and the solid lines for the simulation results. Parameters are the dispersal variance of seeds 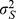 = 0.04 and pollen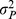 = 0.08, the selection coefficient *s*_1_ = *s*_2_ = 0.02, the relative selection intensity 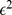 = 1.0, and the recombination rate *r* = 0.1.

Simulations confirm that the analytical model generally performs well for the composite digenic LD (Figure 5A). The maximum composite digenic LD is located at the zone center, and the analytical prediction is slightly greater than the simulation result. Different spatial patterns exist among zygotic LDs, similar to the simulation results in the preceding section. *D_AABB_* has only one positive maximum value (Figure 5B; at the left side *x*<0). *D_AaBB_* has a minimum value (negative) at the left side (*x*<0), denoted by 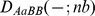, but a small peak (positive) at the right side (*x*>0), denoted by 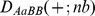. *D_AaBb_* has one peak at the zone center, and the analytical model for *D_AaBb_* predicts one peak distribution across the zone (Figure 5B).

With an increase in dispersal variance, the maximum composite digenic LD predicted from the analytical model is slightly higher than the simulation result. The analytical model performs well for *D_AABB_* and *D_AaBB_*, but are slightly biased for *D_AaBb_*. Double peaks exist in the *D_AaBb_* distribution, but only the one peak is predicted from the analytical model (data not shown here).

**Figure 6:**
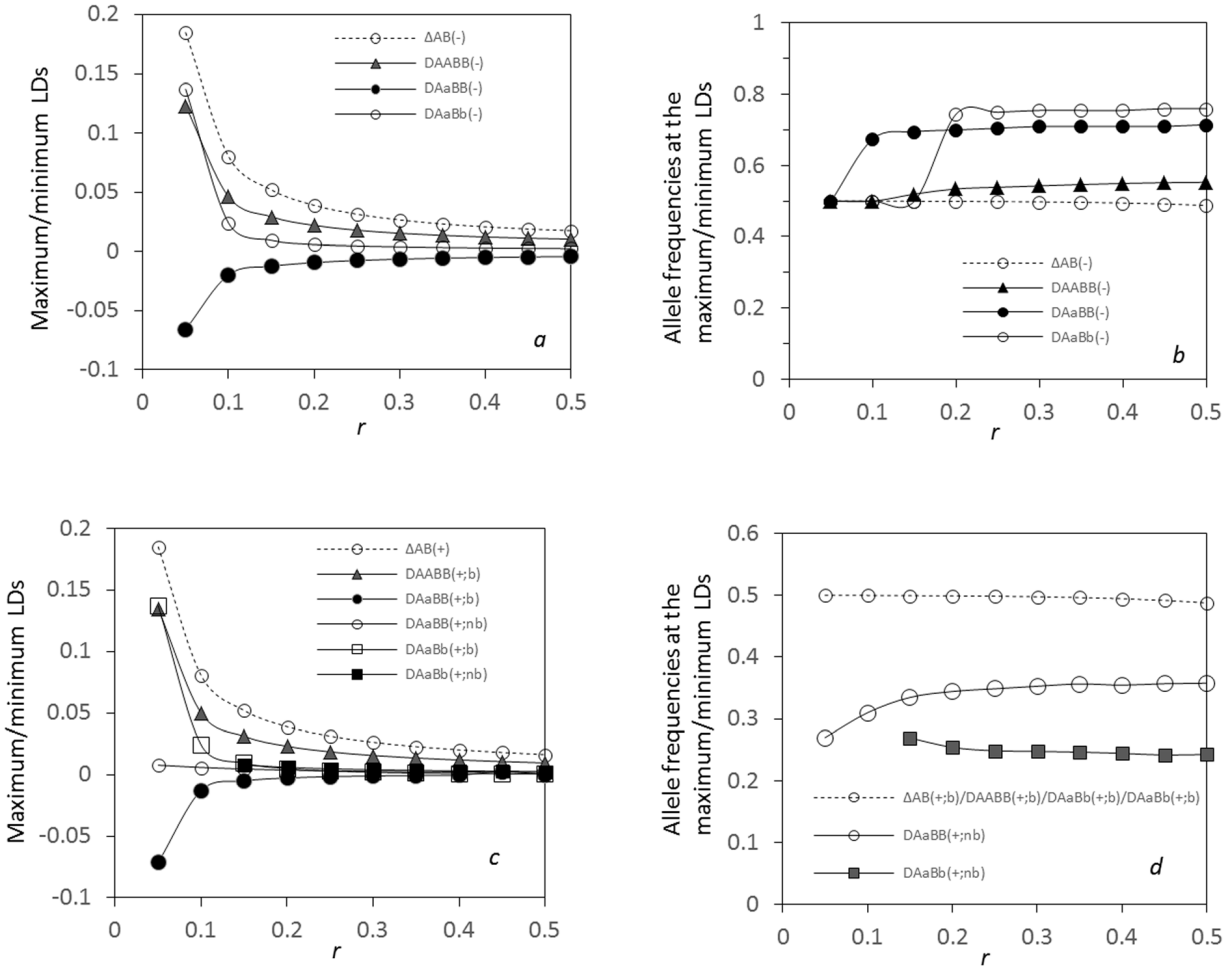
Effects of linkage distance on the maximum or minimum zygotic LDs and the maximum composite digenic LD in an ecological zone: a. the maximum or minimum LDs at the left side (*x*<0); b. the allele frequencies at the positions of the maximum or minimum LDs at the left side (*x*<0); c. the maximum or minimum LDs at the right side (*x*>0); d. the allele frequencies at the positions of the maximum or minimum LDs at the right side (*x*>0). Results are obtained from the analytical model. Parameters are the migration rate of pollen 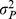 = 0.02 and seeds 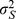 = 0.01, the relative selection intensity 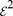 = 1.0, and the selection coefficient *s*_1_ = *s*_2_ = 0.02.

Linkage distance can alter the maximum or minimum zygotic LDs at each side of a hybrid zone. At the left side (*x*<0), the maximum 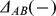 (the boundary point near the zone center) gradually reduces with the recombination rate, and so do other zygotic LDs (Figure 6A). Positions for the maximum or minimum LDs change with the recombination rate (Figure 6B). The maximum 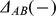 is stably located at the zone center. The position for the maximum 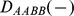 moves to the populations with slightly high gene frequencies. The positions for the minimum 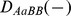 and the maximum 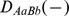 discordantly move to the places with high gene frequencies.

At the right side of the hybrid zone (*x*>0), the maximum composite digenic LD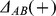 stably occurs at the zone center (Figure 6C). 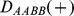 has a maximum stably occurring at the zone center, denoted by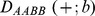, and this value decreases with the recombination rate. 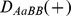 at the boundary point, denoted by 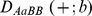, has a minimum value within a short linkage distance (e.g., *r*<0.15 in Figure 6C), and then has a maximum value (>0) at the places with small gene frequencies. This maximum point slightly moves towards to the zone center (Figure 6D). 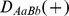 has a maximum at the boundary point under tight linkage, denoted by 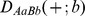, and then has a maximum at the position away from the zone center under loose linkage, denoted by 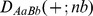.

The above theory indicates that the composite digenic LD displays a robust pattern, with a maximum at the zone center. Zygotic LDs can exhibit diverse patterns in both magnitude and position, depending upon genotypes. The maximum composite digenic LD is always greater than the maximum absolute zygotic LDs under the linear additive selection.

#### Tension zone

From Hu [36], the recursion equation for 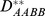 within a time interval, 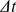, for a population at position *x* is derived as:
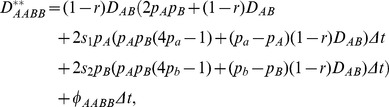
(13)where the migration component 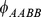 is the same as Eq. (4). The recursion equations for other three zygotic LDs are not detailed further.

Under the coincident clines of gene frequencies, let 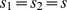, 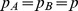, and 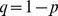. The steady-state zygotic LDs are derived as:
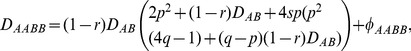
(14)




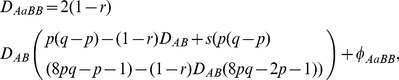
(15)




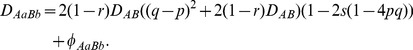
(16)


Again, *D_AaBB_* is equal to *D_AABb_* under coincident clines, but is often unequal to *D_AABb_* under a more general condition (e.g., different selection pressures between loci A and B). It is clear that the selection components in zygotic LDs are different between the ecological and tension zones.


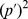
 and 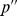 in the migration components, 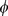
's, are also different from those in the ecological zone: 
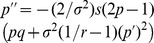
, 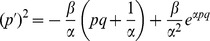 [36].

Under the coincidence of gene frequencies between loci, the steady-state composite digenic LD is derived as
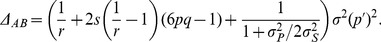
(17)


Since the maximum of 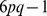
is ½, the maximum *Δ_AB_* is always greater than the maximum gametic LD in the presence of selection and migration. Again, seed flow enhances this difference while pollen flow reduces it.

**Figure 7:**
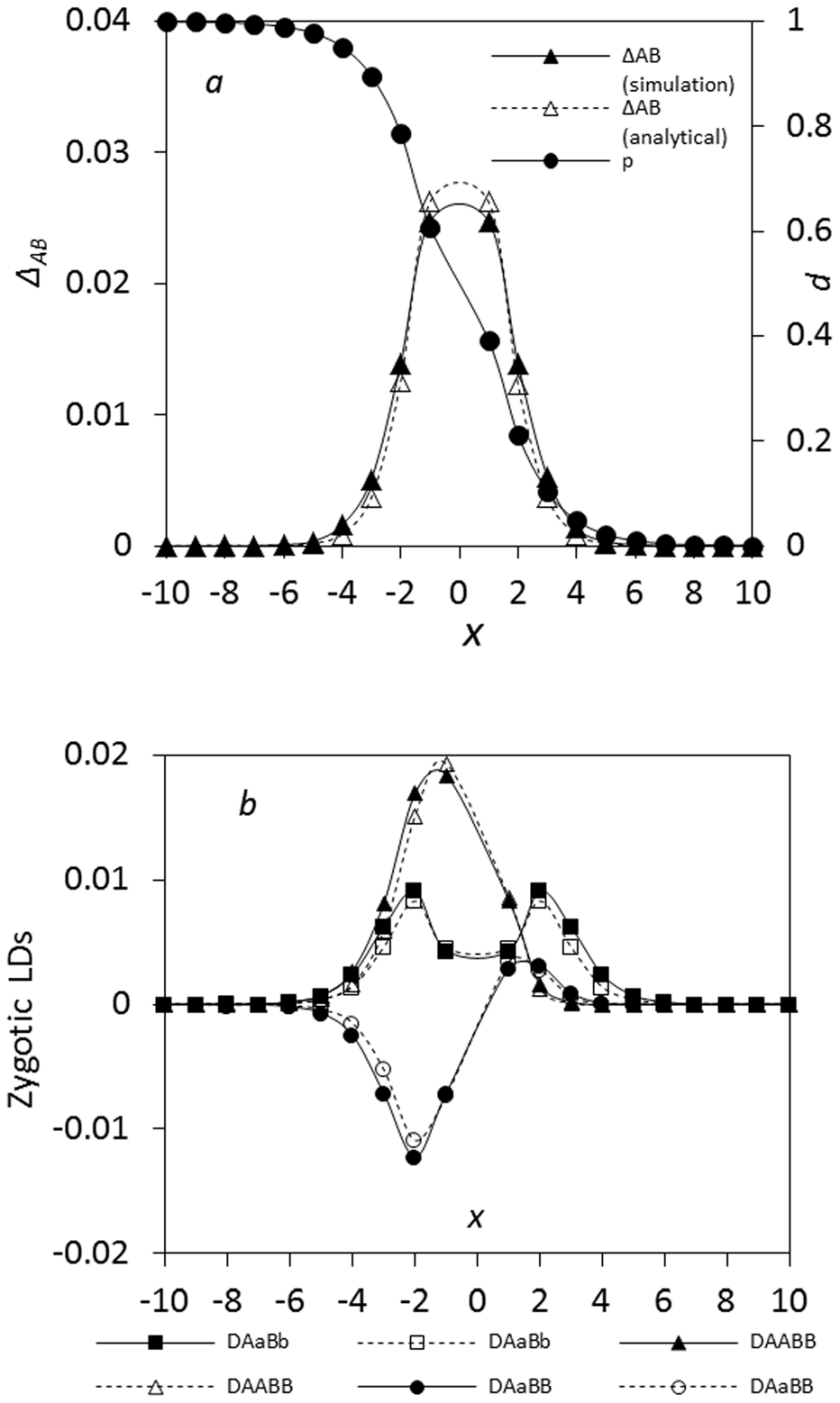
A comparison between the analytical model and simulation results in a tension zone (no genetic drift effects). In (a) and (b), the dashed lines represent the results from the analytic model, and the solid lines for the simulation results. Parameters are the dispersal variance of seeds 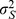 = 0.04 and pollen 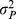 = 0.08, the selection coefficient *s*_1_ = *s*_2_ = 0.02, and the recombination rate *r* = 0.1.

Simulations verify that the analytical model performs well for both the composite digenic and zygotic LDs. The composite digenic LD *Δ_AB_* predicted from the analytical model is slightly greater than the simulation result (Figure 7). It always has a maximum value at the zone center (*p* = 0.5), which can also be analytically proven from 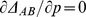 since it is the function of *pq* from Eq. (17). The spatial patterns of zygotic LDs are essentially similar to those in the ecological zones (Figure 7B). *D_AABB_* has a maximum value at one side (*x*<0). *D_AaBB_* has a minimum value (negative) at one side (*x*<0) but a small peak (positive) at the other side (*x*>0). *D_AaBb_* has two symmetric peaks (positive) across the zone, with one peak at each side of the zone center. These maximum and minimum zygotic LDs are located in different spatial positions.

**Figure 8:**
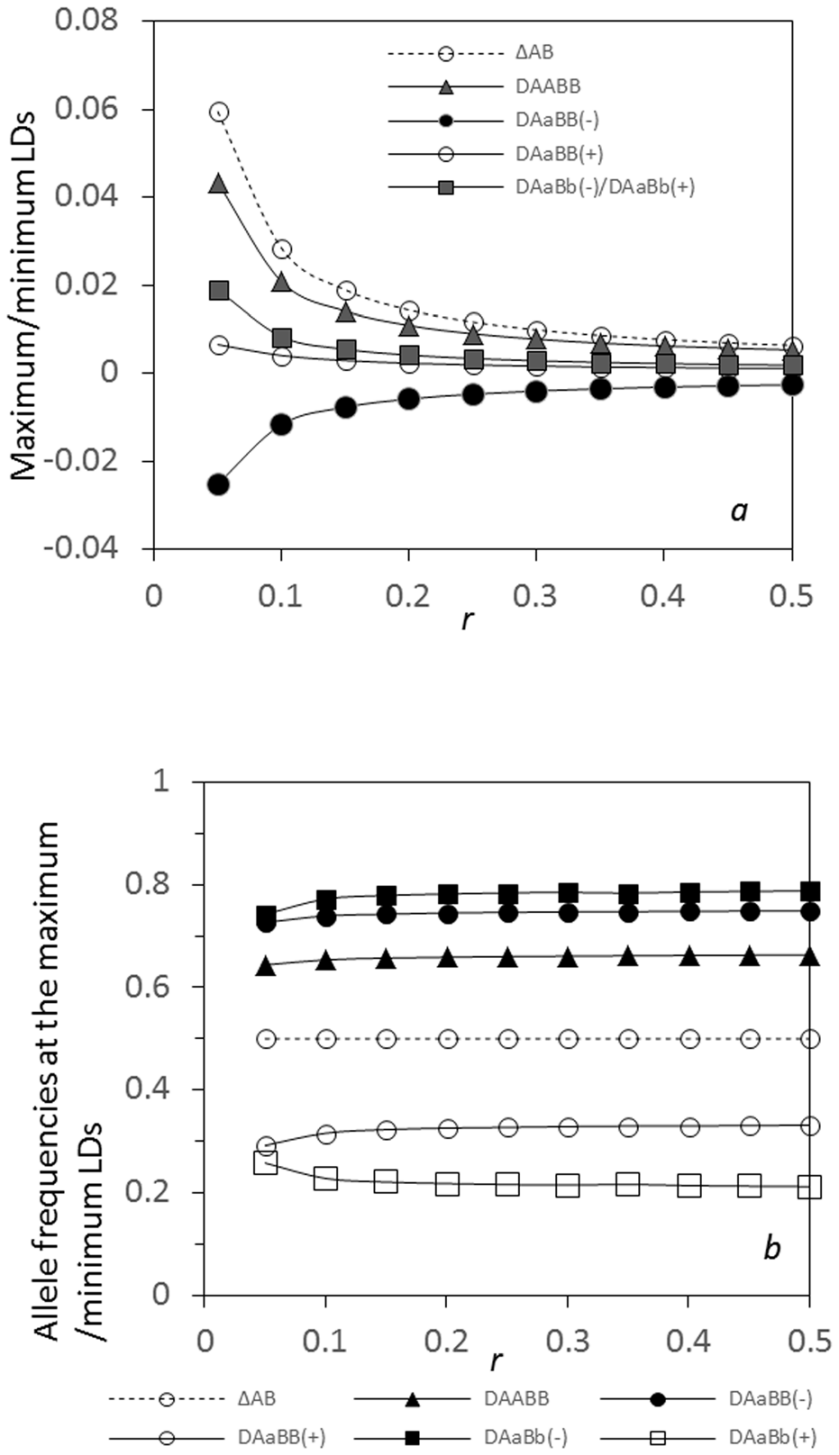
Effects of linkage distance on the maximum or minimum zygotic LDs and the maximum composite digenic LD in a tension zone: a. the maximum or minimum LDs; b. the allele frequencies at the positions of the maximum or minimum LDs. Results are obtained from the analytical model. Parameters are the migration rate of pollen 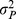 = 0.02 and seeds 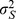 = 0.01, and the selection coefficient *s*_1_ = *s*_2_ = 0.02.

The maximum composite digenic LD *Δ_AB_* is always greater than the maximum zygotic LD in magnitude. Figure 8A indicates that the maximum composite digenic (*Δ_AB_*) and zygotic LDs gradually decrease as the recombination rate increases, including 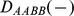 (at the side of x<0 only), 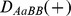 (at the side of *x*>0), and 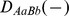 or 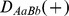 (at both sides of *x* = 0). The minimum zygotic 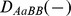 (negative; at the side *x*<0) gradually decreases with the recombination rate. Their separate spatial positions are relatively stable (Figure 8B).

### Application to a Mouse Hybrid Zone

#### Materials

The preceding theory can be applied to animal hybrid zones by removing pollen flow and replacing the seed flow with animal dispersal. In this section, we apply this theory to analyzing a house mouse hybrid zone (*Mus musculus* and *M*. *domesticus*). Genotyping data of this hybrid zone are publically obtained from the supplementary data of Teeter et al. [9] (http://genome.cshlp.org/content/suppl/2007/11/19/gr.6757907.DC1.html; Supp Table2.doc and Supp Table4.xls). The SNP markers are located on the whole mouse genome: 39 autosomal SNP markers (3 on chromosomes 1 and 2, 1 on chromosome 16, 2 on each of the rest 16 chromosomes), and 13 SNP markers on X-chromosome. The mouse hybrid zone is a tension zone [9], as indicated in a separate study [38]. The hybrid zone was formed in Western Europe within the last 3000 years, and the focal zone examined by Teeter et al [9] is in Bavaria, Germany. On the basis of the composite digenic LD pattern in the zone center population (Neufaham bei Freising), Teeter et al. [9] concluded that epistatic interactions (Dobzhansky-Muller incompatibility model) occurred in this hybrid zone.

For the autosomal SNP pairs, thirteen populations are investigated, each with the sample size of not less than 10 individuals (albeit this size remains small). These populations are Augsburgh (Locality 1), Appercha (7), Gesselthausen-Warta (8), Gesselthausen-Ziigletrumm (9), Eberspoint (11), Massenhausen/Neufahrn (12), Neufahrn bei Freising (14), Achering (18), Rudlfing (22), Schwaig (23), Sonnendorf (26), Brundl (29), Simbach (32), and one population Leitham-Fuchs (40) in Austria. For the X-chromosome SNP pairs (we treat all females as a subpopulation; [47]), and the SNP pairs between X-chromosome and autosomes, we use 10 populations each with more than 10 females (without Localities1, 11, and 12 in the preceding list of populations). Only females are used for analysis since the theory deals with the diploid genotyping data.

#### Statistical methods

The maximum likelihood estimate (MLE) of zygotic LD, *D_AABB_*, can be obtained by
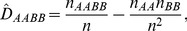
(18)where 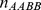, 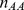, and 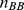 are the numbers of genotypes *AABB*, *AA*, and *BB*, respectively, and *n* is the sample size. Using Fisher's method ([16], p.126), we can derive the large-sample variance of the zygotic LD:
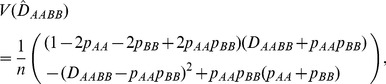
(19)where 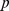
's in Eq. (19) are the genotypic frequencies estimated from the sample. The expected MLE 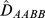 over replicate samples of *n* individuals from the same population, 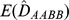, is derived as
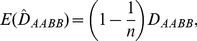
(20)indicating that the estimate 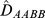 is biased. As the sample size *n* approaches sufficiently large, 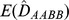 equals *D_AABB_*.

For the large sample sizes, the MLE 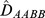 approximately follows a normal distribution, and a normalized zygotic LD is constructed as
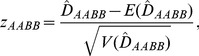
(21)which can be used to test the null hypothesis H_0_: *D_AABB_* = 0. 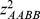 follows a chi-square distribution with one degree of freedom. Thus, an equivalent chi-square statistic
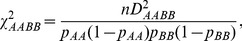
(22)is constructed to test the null hypothesis H_0_: *D_AABB_* = 0 ([22], p.441).

Replacing subscripts *AABB*, *AA*, and *BB* in Eqs. (18) to (22) with subscripts *AaBB*, *Aa*, and *BB*, yields the MLE of *D_AaBB_*, *V*(*D_AaBB_*), *z_AaBB_*, and 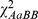, respectively. Replacing subscripts *AABB*, *AA*, and *BB* in Eqs. (18) to (22) with subscripts *AABb*, *AA*, and *Bb*, yields the MLE of *D_AABb_*, *V*(*D_AABb_*), *z_AABb_*, and 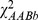, respectively. Replacing subscripts *AABB*, *AA*, and *BB* in Eqs. (18) to (22) with subscripts *AaBb*, *Aa*, and *Bb*, yields the MLE of *D_AaBb_*, *V*(*D_AaBb_*), *z_AaBb_*, and 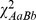, respectively. These z-scores and chi-squares are applied to testing the significance of different zygotic LDs.

The MLE 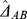 can be obtained by the counting method according to Eq. (2). Its sampling variance is derived using Fisher's method ([16], p.50),
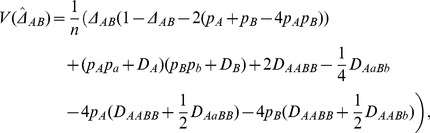
(23)where 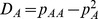 and 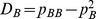 are the HWD at loci A and B, respectively. The expected MLE over replicate samples of *n* individuals is derived as 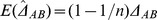, which is biased. Thus, a normalized parameter can be constructed to test H_0_: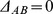, 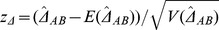, and z*_Δ_* follows a normal distribution for the large sample sizes. An equivalent chi-square statistic, the same as the distribution of 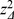, is 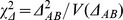 under the null hypothesis H_0_: 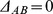.

To test 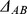, we follow Weir and Cockerham's [42] suggestion by firstly testing each of the four independent zygotic LDs. The insignificant zygotic LD is then dropped. If all zygotic LDs are insignificant from zero, the test statistic recovers the expression 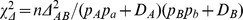 [42].

To test the difference between composite digenic and zygotic LDs, we consider two cases. When both *Δ_AB_* and *D_AABB_* are positive or negative, let 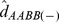 be the MLE of the difference between the composite digenic LD and the zygotic LD for one parental genotype ( = 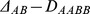). Using Fisher's delta method, we obtain the variance of the MLE, 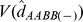,
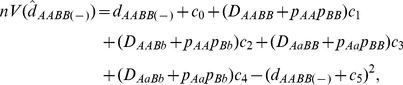
(24)where *c* coefficients are given in Table 1. The expected MLE over replicate samples from the sample population is derived as 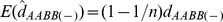. For the large sample sizes, 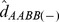 follows a normal distribution, with the mean 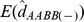 and variance 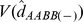. A normalized parameter,
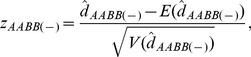
(25)can be constructed to test H_0_: *d_AABB_*_(-)_ = 0.

**Table 1:** Coefficients for calculating the variances of the differences between composite digenic and zygotic LDs.

Differences	*c* coefficients
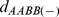	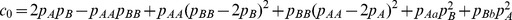
	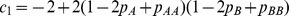 ; 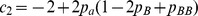
	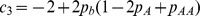 ; 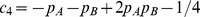 ; 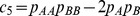
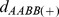	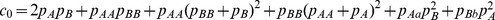
	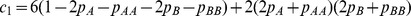 ; 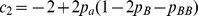
	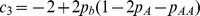 ; 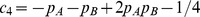 ; 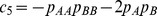
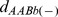	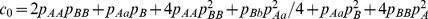 ; 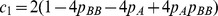
	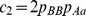 ; 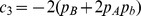 ; 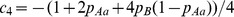 ; 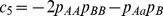
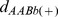	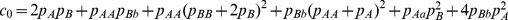
	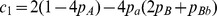 ; 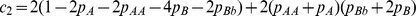
	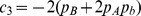 ; 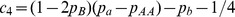 ; 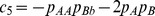
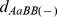	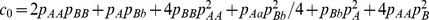 ; 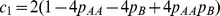
	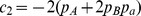 ; 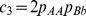 ; 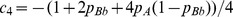 ; 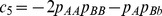
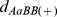	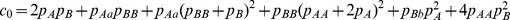
	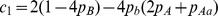 ; 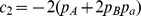
	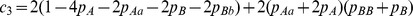
	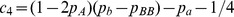 ; 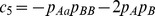
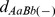	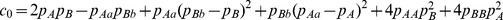 ; 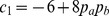
	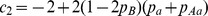 ; 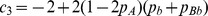
	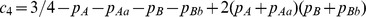 ; 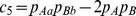
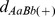	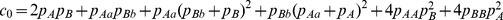 ; 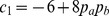
	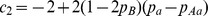 ; 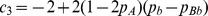
	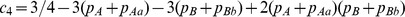
	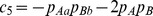

An equivalent chi-square test statistic with one degree of freedom, the same as the distribution of 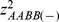, is
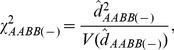
(26)for testing the hypothesis H_0_: *d_AABB_*_(-)_ = 0 [42].

When *Δ_AB_* is positive but *D_AABB_* is negative, or vice versa, the difference between can be set as 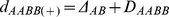. Its variance 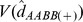 can be obtained by replacing 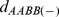 in Eq. (24) with 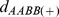 and using the corresponding *c* coefficients given in Table 1. 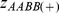 and 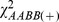 are obtained by replacing 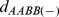 in Eqs. (25) and (26) with 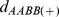, respectively.

Similarly, let 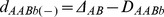, 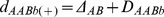, 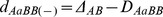, 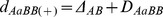, 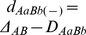, and 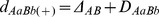 for other comparisons. Their variances can be obtained by replacing subscript *AABB*(-) in Eq. (24) with different subscripts, and the corresponding *c* coefficients are given in Table 1. The test statistics can be obtained by replacing the subscript *AABB*(-) in Eqs. (25) and (26) with different genotypes to test the significance of these differences. Again, following Weir and Cockerham's [42] suggestion, we firstly test individual zygotic LDs. Insignificant zygotic LDs are then dropped in testing *d*'s.

The above approaches are used to test the composite digenic and zygotic LDs, and the difference between the maximum composite digenic LD and the maximum zygotic LD, i.e. max. |*Δ_AB_*|-max.|
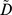
|, in which 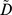 refers to one of the four zygotic LDs (*D_AABB_*, *D_AABb_*, *D_AaBB_*, *D_AaBb_*). Both the normalized *z* and χ^2^ tests produce the same results for any pair of SNPs, and duplicated results are not shown below. To reduce the influences of rare alleles on LD estimations, we have removed the SNP markers whose allele frequencies are smaller than 5% or greater than 95%. Statistical analyses include HWD test for each SNP using chi-square statistic ([16], pp. 96–97).

Note that all the above tests differ from the existing methods in that they directly rely on genotypic data, without the need of estimating gametic LD from diploid genotypes [16], [28], [42]. This alternative approach is applicable to any natural population with an arbitrary mating system.

#### Empirical results

HWD tests are summarized in Supplementary S1. A majority of SNPs on autosomes exhibited HWE in most populations except the population at Locality 32. Those SNPs with HWD had significant heterozygote deficit. A few SNPs exhibited HWD simultaneously in multiple populations with either large or small sample sizes, such as SNPs 2.03, 13.056, 19.044, X.033, and X.099a. Almost all these SNPs were involved in significant zygotic LDs or significant composite digenic LD (Supplementary S2). Population at Locality 32 exhibited extensive HWD (16 out of 39 autosomal SNPs, and 8 out of 13 X-chromosomal SNPs). Reasons for HWD at Locality 32 are unclear to us, and other processes besides genetic drift could also be responsible for this pattern since most SNPs exhibited HWE even in the small populations.

Tests of four independent zygotic LDs and *Δ_AB_* are summarized in Supplementary S2. There were significant composite digenic LD or zygotic LDs in 13 localities for autosomal SNP pairs, 2 localities for X-chromosomal SNP pairs, and 7 localities for the pairs between autosomal and X-chromosomal SNPs. These SNPs exhibited various patterns of non-random associations across the hybrid zone. On the basis of Teeter et al. [9], several additional results are obtained here. The first result is that the composite digenic LD cannot appropriately indicate the genotypic associations that are potentially associated with postzygotic isolation. For the autosomal SNP pairs (599 in Table 2 derived from Supplementary S2), there were 61 pairs (10.1%) with significant composite digenic LD but insignificant zygotic LDs, 98 pairs (16.4%) with significant composite digenic LD and at least one significant zygotic LD, and 440 pairs (73.5%) with insignificant composite digenic LD but significant zygotic LDs. For the X-chromosomal SNP pairs, there were 6 pairs (8.7%) with significant composite digentic LD but insignificant zygotic LDs, 19 pairs (27.5%) with significant composite digenic LD and at least one significant zygotic LD, and 44 pairs (63.8%) with insignificant composite digenic LD but significant zygotic LDs. For the pairs between autosomal and X-chromosomal SNPs, there were 12 pairs (4.3%) with significant composite digenic LD but insignificant zygotic LD, 38 pairs (13.6%) with significant composite digenic LD and at least one significant zygotic LD, and 229 pairs (82.1%) with significant zygotic LDs but insignificant composite digenic LD. In general, a majority of significant zygotic LDs, 75.3%, cannot be reflected from the pattern of composite digenic LD.

**Table 2:** Summary of statistical tests (Supplementary S2) for the composite digenic and four independent zygotic LDs and their differences in a house mouse hybrid zone (*Mus musculus* × *M*. *domesticus*).

Combinations	Autosomes	X-Chromosome	Autosomes and X-Chromosome	Total
Sig. *Δ_AB_* and sig. 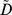	98(16.4%)	19(27.5%)	38(13.6%)	155(16.4%)
Sig. *Δ_AB_* and insig. 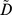	61(10.1%)	6(8.7%)	12(4.3%)	79(8.3%)
Insig. *Δ_AB_* and sig. 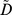	440(73.5%)	44(63.8%)	229(82.1%)	713(75.3%)
Max. |*Δ_AB_* | = max. | 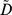 |	569(94.8%)	56(81.2%)	268(96.4%)	893(94.3%)
Max. |*Δ_AB_* |<max. | 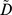 |	20(3.3%)	0	3(1.1%)	23(2.4%)
Max. |*Δ_AB_* |>max. | 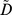 |	11(1.8%)	13(18.8%)	7(2.5%)	31(3.3%)

The second result is about the relative extents of the composite digenic and zygotic LDs (Table 2 and Supplementary S2). Generally, about 94.3% of SNP pairs had their composite digenic LDs that were comparable to the maximum zygotic LDs, i.e. max. |*Δ_AB_*| = max. |
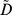
|, indicating the presence of potential epistatic selection among these loci. These include SNP pairs from autosomes, X-chromosome, or the pairs between autosome and X-chromosome, such as the genotypic association from the same chromosome (e.g., SNP 4.057 and 4.129 in Locality 14; *Δ_AB_* = 0.0505*** (p<0.0001), *D_AABb_* = 0.0744* (p<0.05), and |*D_AaBb_* | = 0.076** (p<0.01)) or from different chromosomes (e.g., SNP15.099 and 17.046 in Locality 14; *Δ_AB_* = 0.0525***, *D_AABb_* = 0.0774*, and |*D_AaBb_*| =  0.0696*). The composite digenic LD mainly arises from the interactions of alleles from separate chromosomes. About 2.4% of all SNP pairs showed strong epistatic selections occurring among these loci, i.e. max. |*Δ_AB_* |<max.|
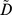
|, and this mainly occurred for the SNP pairs from autosomes (3.3%) or between X-chromosome and autosomes (1.1%). Epistatic selection (e.g., Dobzhansky-Muller's incompatibility) was more likely involved in their genotypic interactions. A small proportion of SNP pairs (3.3%) possess significantly larger composite digenic LD than the maximum zygotic LD, i.e. max. |*Δ_AB_*|>max. |
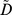
|, indicating that the linear additive selection occurred among these SNP pairs. This occurred for SNP pairs from autosomes (1.8%), X-chromosomes (18.8%), and between X-chromosome and autosomes (2.5%). Thus, the linear-additive selection process is also potentially associated with postzygotic isolation in this mouse hybrid zone.

**Figure 9:**
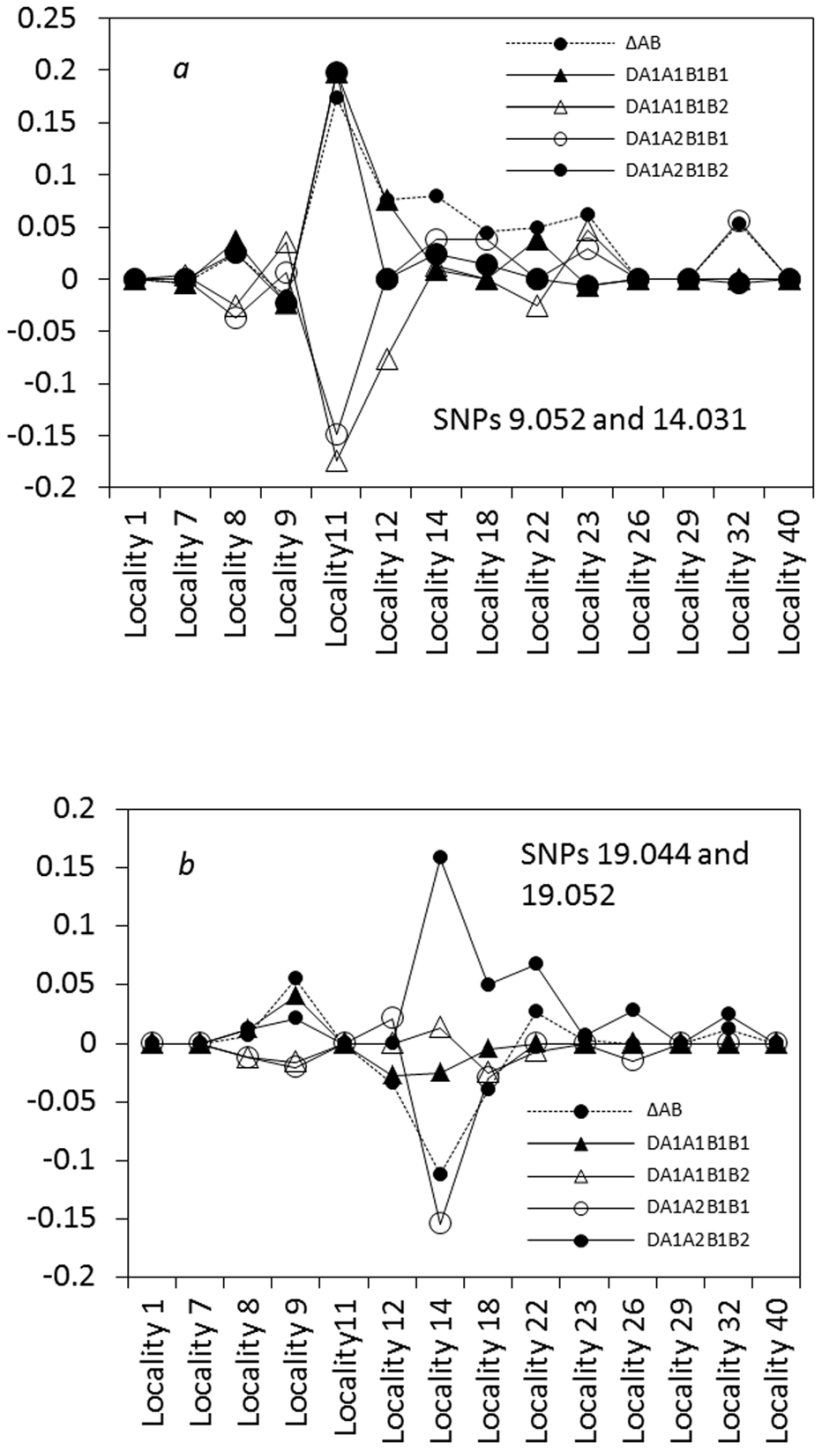
Distribution of the composite digenic and zygotic LDs across a house mouse hybrid zone (*Mus musculus* × *M*. *domesticus*; [9]): a. the genotypic association for SNP pair 9.052 and 14.031; b. the genotypic association for SNP pair 19.044 and 19.052. In (a), three zygotic LDs are significant but the composite digenic LD is insignificant at Locality 11. In (b), two zygotic LDs and the composite digenic LD are significant at Locality 14 (Supplementary S2).

The third result is the discordant patterns between the composite digenic and zygotic LDs across the hybrid zone (Supplementary S2). Some significant zygotic LDs were located at the zone center (Locality 14), but many significant zygotic LDs also occurred outside the central regions (e.g., Locality 9). The maximum or minimum zygotic LDs and the maximum composite digenic LD occurred at different positions. These support the asymmetric gene introgression between *Mus musculus* and *M*. *domesticus* [9], [38]. For instance, a genotypic association from different chromosomes, SNP pair 9.052 and 14.031 (max. |*Δ_AB_*| = max.|
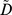
|), had the maximum composite digenic and zygotic LDs in magnitude at Locality 11(the left side of the zone center; Figure 9a), indicating an asymmetric spread of SNPs 9.052 and 14.031 between these two species. A few genotypic associations from the same chromosome had significant maximum composite digenic and zygotic LDs at Locality 14 (zone center), such as SNPs 19.044 and 19.052 (|*Δ_AB_*| = 0.1119**, |*D_AaBB_*| = 0.1540** and *D_AaBb_* = 0.1582**, but max. |*Δ_AB_*| = max. |
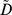
|; Figure 9b). For these SNPs, their genotypic interactions produced by epistatic selection at the zone center could effectively act as a biological barrier to gene introgression to each species.

There were many significant zygotic LDs (mainly, one parental *D_AABB_*) at Locality 32 for the SNP pairs from autosomes, X-chromosome, or between autosome and X-chromosome (Supplementary S2). No significant zygotic LDs existed in recombinant genotypes. The reason besides the genetic drift effects remains unclear, and is probably associated with the asymmetric gene flow where the recombinants derived from the fusion of immigrants with residents occurred more recently.

## Discussion

In this study, we propose the use of zygotic LD to characterize genotypic interactions and compare its pattern with that of the composite digenic LD in a hybrid zone where two genetically diverging species are partially integrated through hybridizations. We analytically associate the composite digenic and zygotic LDs with the liner-additive selection process. It is clear that the composite digenic LD measures the non-random associations of two alleles (one allele from each locus), and can be affected by selection at either the gametophyte stage or sporophyte stage, or both. Zygotic LD measures the non-random associations of four alleles (two alleles from each locus) at the sporophyte stage only and directly relates to the potential postzygotic isolation. Both types of measures can be applied to naturally hybridizing populations where HWE or random mating is violated. It is commonly believed that a low-order LD is stronger than a high-order LD for a given pair of loci. We demonstrate that this is true in the liner-additive-viability model, but does not hold under the epistatic selection. This provides a theoretical basis for using the joint patterns of the composite digenic and zygotic LDs to elucidate the selection mechanisms of postzygotic isolation (a liner-additive-viability model versus epistatic selection). The empirical results from a house mouse hybrid zone evidence that the composite digenic LD cannot fully characterize genotypic interactions because it confounds the information from multiple zygotic LDs. Also, a part of significant zygotic LDs cannot enable a significant composite digenic LD. Thus, a sole reliance on HWD test or the composite digenic LD cannot elucidate the type of underlying selection process in a hybrid zone.

Also, a reliance on the joint patterns of HWD and gametic LD cannot explicitly reveal any genotypic interactions that are potentially associated with postzygotic isolation. HWD may arise from single or multiple processes that are irrelevant to genotypic interactions, including the effects of gene flow, drift, mating system, and selection in either gametic or zygotic stage at a single locus. It may also arise from selection at the linked loci via associative overdominance or genetic hitchhiking process, which might be relevant to the genotypic interaction. This complexity makes it difficult to detect genotypic interaction with HWD. A similar situation exists with the gametic LD analysis where single or multiple evolutionary processes are involved. Although gametic LD measures a general non-random association between two loci, it does not specify genotypic interactions. Further, practical gametic LD analysis needs the assumption of random mating or HWE, which is often violated in naturally hybridizing populations. Thus, it is inappropriate to use gametic LD together with HWD to infer postzygotic isolation produced by genotypic interactions.

Since the composite digenic or gametic LD measures a general non-random association between two loci, only one normalized parameter (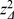 or 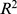) is needed, without the information of genotypes and linkage phases. Test based on 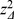 or 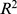 does not reveal specific genotypic interactions. To apply the proposed theory to detecting selection model, four normalized parameters (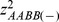 or 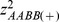, 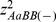 or 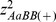, 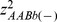 or 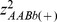, and 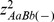 or 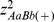) are needed for a pair of diallelic loci, the most common case with SNP markers since tri- or tetra-allelic SNP markers are infrequent in natural populations. These individual normalized parameters are specific to two-locus genotypes, different from 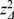 that confounds the information of four independent zygotic LDs. One caution is that only the normalized parameter or the chi-square of the two-locus genotype with the maximum absolute zygotic LD is used to infer selection model (additive versus epistatic selection). From the theoretical results, this stringent test enables a more conservative inference on epistatic selection.

Zygotic LD is more informative than the composite digenic LD on the potential evolutionary processes in the transient phase of speciation, which is similar to the conclusions in a continent-island model of population structure [32]. The composite digenic LD displays only one pattern of distribution across a hybrid zone, with a maximum in the zone center, similar to the pattern of gametic LD [41]. Zygotic LD has various patterns, depending upon specific genotypes. It can exhibit the distribution similar to that of the composite digenic LD, or a two-peak distribution, or a distribution contrasting to that of the composite digenic LD. Zygotic LDs for the parental genotypes (*AABB* and *aabb*) exhibit one-peak distribution across a hybrid zone. Zygotic LDs for the homozygote-heterozygote genotypes display one minimum value at one side of the zone and a maximum at the other side. Although we investigate the coincident zygotic LDs between two homozygote-heterozygote genotypes (e.g., *D_AaBB_* versus *D_AABb_*, or *D_Aabb_* versus *D_aaBb_*) by the symmetric parameter settings, their non-coincidence may indicate unequal gene introgression at individual loci. This can be produced by different processes, including the distinct selection pressures between genotypes *Aa* and *Bb*, the distinct genotypic interactions between heterozygote at one locus and homozygote at the other locus, and the asymmetric gene flow between loci. Similarly, a complex process is also involved in changing the pattern of the zygotic LD for the heterozygote-heterozygote combination. The pattern can be used to infer the position of maximum genotypic interaction and the pattern of gene introgression.

It is well understood that natural hybrid zones provide an excellent natural laboratory to study the mechanisms of postzygotic isolation [45], [48], [49]. Barriers to gene introgression are expected to be greater for the genes associated with reproduction isolation than for the neutral genes [49], [50]. The selection process (intrinsic versus extrinsic; linear-additive versus epistatic) plays a critical role in impeding postzygotic gene introgressions in natural hybrid zones [51], [52], [53]. This consequently forms different blocks of introgression along chromosomes in each species [12], [13]. Genotypic frequencies are not often directly applied to inferring the mechanism of hybridization zone. Instead, the gametic LD is used to predict the barrier to gene flow or the strength of selection [15], [48], [49], which requires the assumption of HWE or random mating. The present study shows the usefulness of using zygotic LD in conjunction with the composite digenic LD to reveal the selection model of genotypic interactions. With the application of genome-wide SNPs across a hybrid zone, such analyses could generate a network where the selection model of genotypic interactions can be annotated. This may give us a comprehensive picture of understanding the mechanism of postzygotic isolation [54].

Although the composite digenic and zygotic LDs are genetically related because both of them are the function of gametic LD (e.g., see the analytical theory), their differences are clear in terms of the selection components. The composite digenic LD can arise from a mixture of multiple selection effects on hybrids, including (i) the additive selection effects from individual loci at the gametophyte stage, (ii) the cumulative dominance effects from individual loci at the sporophyte stage, (iii) the interaction of dominance by dominance at the sporophyte stage (*D_AaBb_*); (iv) the interaction of dominance by additive effects at the sporophyte stage(*D_AaBB_* or *D_AABb_*); and (v) the interaction of additive by additive effects at the sporophyte stage (*D_AABB_*). The first two selection effects are related to the linear-additive-viability model while the later three selection effects are related to epistatic selection. Since multiple selection effects are involved in changing the composite digenic LD, it is difficult to elucidate the principle selection model solely based on the pattern of digenic LD. This necessitates the analysis of individual zygotic LDs where the potential selection model can be specified once the selection model (linear-additive selection versus epistatic selection) is determined.

Previous studies mainly concentrate on the connection of gametic LD to the mechanism of reproductive isolation in hybrid zones [55]. Although both gametic and zygotic LDs are correlated in statistics due to sampling [20], [22], they are different in connection to ecological and evolutionary processes (a functional but not a statistical relationship; [32]). Like the composite digenic LD, additional information is needed to infer the underlying selection processes if the pattern of gametic LD is used.

It is also clear that the proportions of zygotic and composite digenic LDs generated by gene flow and genetic drift are essentially not related to the functional genotypic interactions. These parts can be considered as the background variation in detecting the selection process in the dispersal-dependent hybrid zones, analogous to the null hypotheses in a statistical test. However, the relationships between zygotic and composite digenic LDs are not purely statistical relationships since evolutionary processes are involved. The remaining issue is that weak additive or weak epistatic selection could slightly modify such background spatial patterns of the composite digenic and zygotic LDs, and hence could be hard to detect in practice. The pattern-based comparisons, including the relative maximum composite digenic and maximum zygotic LDs and their spatial distribution patterns, may be difficult to test natural selection model in a hybrid zone. How large sample sizes are needed to detect weak epistatic selection forms an important topic for further study.

Although the theory is developed under the presence of gene flow in space, the theoretical conclusions can be applied to the completely isolated population or the admixture of artificial populations. Gene flow increases both the composite digenic and zygotic LDs, but does not change their relative patterns under strong epistatic selection between genotypes. Genetic drift effects do not change their relative patterns as well. Comparing the difference between the maximum composite digenic LD and the maximum zygotic LD can aid in inferring if epistatic selection exists between genotypes, respective of the pattern of mating system. Also, the theoretical conclusions can be applied to genome-wide screening for the SNPs exhibiting genotypic rather than gametic epistasis for populations under distinct environments (e.g., disease infected vs uninfected populations). This is a useful approach to detect genetic epistasis at the diploid level, alternative to the quantitative traits-based approaches [56], [57], such as detection of epistasis in genome-wide association studies (GWAS).

Finally, it is of interest to compare the pattern of composite digenic and zygotic LDs under the linear-additive-viability model in either a tension zone or an ecological zone. One feature is a two-peak distribution for *D_AaBb_* across a hybrid zone under a symmetry gene introgression in two directions for loosely linked loci. More tightly linked loci (or increasing selection coefficient) can make these two-peaks towards the zone center. However, one-peak pattern for *D_AaBb_* may occur under asymmetric introgression or only one-way introgression for loosely linked or unlinked loci. When the maximum *D_AaBb_* is on the left side of the zone center, the gene introgression from the species on the right side could be more extensive across the hybrid zone and vice versa. In flowering plants, differential reproductive systems between species often cause unequal pollination rates and seed dispersal rates. Gene introgression is expected to be greater for the more outcrossing species. Also, pollen flow is often much greater than seed flow for the more outcrossing species [58], [59], enhancing asymmetric gene flow. Under this situation, it is speculated that a frequent outcome could be the one-peak pattern for *D_AaBb_* across a natural hybrid zone.

## Supporting Information

Supplementary S1 Hardy-Weinberg disequilibrium tests.
(XLSX)

Supplementary S2 Composite digenic and zygotic LDs in a mouse hybrid zone.
(XLSX)
